# Intelligence

**Published:** 1831-08

**Authors:** 


### INTELLIGENCE.

### MONTHLY REPORT OF DISEASES.

In consequence of the dreadful and well-authenticated accounts which have been transmitted to this country of the extensive ravages which cholera has been making in various parts of Russia, much apprehension has existed in the public mind upon the subject; and cases which at other periods would have caused no alarm, have been regarded with the utmost suspicion and dread. It is very true that, during the last month or six weeks, there has been, both in the metropolis and in various parts of the country, an unusual prevalence of disease; but, as far as we know, and we have taken much pains to ascertain the truth, cholera has not been more frequent or more severe than it generally is at this season of the year. We are not aware that any one instance has occurred in this country of the malignant and very fatal disease which has committed such havock in the north of Europe.

Dysentery and Diarrhœa have been, and still continue, frequent: the cases we have seen were not severe.

Epidemic catarrh, or Influenza, has been the most prevailing malady: in the course of the last month, upwards of thirty cases have fallen under our own observation. In a few instances the symptoms were very severe, but in general the disease has been mild in its nature, and easily removed by ordinary treatment. The influenza of the present moment differs so little from that described by Sydenham and many other writers, that we do not deem it necessary to enter into a particular account of it. The most striking feature of the disease is an extreme tendency to depression of the vital powers, and consequently an evacuant treatment, either by bleeding or free purging, must be employed with much caution. Even when the age and general health of the patient have appeared to indicate the propriety of a lowering treatment, it has been found essentially necessary to bear in mind the peculiar disposition of all diseases of the present season to run rapidly into a state of sinking and exhaustion.

---

### *Claim of* Dr. O'Shaughnessy *to the Discovery of a Mode of distinguishing between the Meconate and Sulphocyanate of Iron.*

We have much pleasure in giving insertion to the following letter, and we cannot refrain from taking this opportunity of expressing our sincere admiration for the great zeal and talent which Dr. O'S. has evinced in various important investigations that he has undertaken.

*To the Editor of the London Medical and Physical Journal.*

Sir: My attention has this moment been directed to the series of prize answers by Mr. David William Nash, inserted in the last number of your Journal, in reply to the questions on Materia Medica and Therapeutics proposed by Dr. A. T. Thomson, at the London University. In one of these I find that Mr. Nash erroneously attributes to Dr. Glendinning the discovery of a mode of distinguishing between the meconate and sulphocyanate of iron, a problem of much importance in medico-legal analysis.

That mode of discriminating between the solutions of these two salts, I first pointed out in a paper on the Sulphocyanic Acid, published in the Lancet last October; and Dr. Glendinning, in his valuable Lecture on Opium, subsequently inserted in the same periodical, while speaking of the mode of detecting that poison, quoted the additional observations I had made on the process, with a candour and liberality which ever distinguish men of really eminent acquirements.

From this lecture, or my paper, could Mr. Nash have alone derived his information on the improvement in question; and as Dr. Glendinning prominently acknowledged me to be the author, I am entitled to regard Mr. Nash's suppression with feelings of some surprise.

I shall deem the insertion of this note in your next number to be a mark of particular kindness. In the mean time, I have the honour to be,

Yours very respectfully,

W. B. O'SHAUGHNESSY, M.D.

*Albany road; 22d July.*

---

Dr. ROGET. At the last quarterly meeting of the College of Physicians, Dr. ROGET was elected a Fellow.

---

MIDDLESEX HOSPITAL. An Assistant Surgeon is about to be appointed to this institution. The only candidates who have as yet started are, we believe, Messrs. ARNOTT, PERRY, and STAFFORD.

---

ROYAL WESTMINSTER OPHTHALMIC HOSPITAL, *Chandos street, Charing-cross.*

The annual meeting of the governors of this hospital took place on June 29th, 1831, in the vestry room of St. Martin's parish, for the purpose of receiving the Report of the Committee of Management, and of laying the first stone upon the foundations: the DUKE of RICHMOND, K.G., in the chair.

Sir J. SWINBURNE, in presenting the Annual Report, said that he had, for some years past, taken a great interest in this charity; that he had frequently attended, and seen the whole of the poor persons who applied for relief, and that nothing could exceed the kindness and attention paid to them by all the gentlemen and students of the hospital, and that the number was so great that they could not enter the house, but were obliged to stand in the street. He wished particularly to draw the attention of the governors and the public to the fact that, every year, there were from eighteen to twenty children brought to the hospital, who had lost either one or both eyes, by a disease contracted within the first three weeks of their birth. The names and addresses of them were published in the Report; and if they had been brought at an earlier period, he was sure, from what he had seen in other cases, that they would have been saved from a misery only to be relieved by death. The number who applied in a state of destitution from blindness, although otherwise able to work, was very great; and if the hospital was capable of receiving these poor people, the greatest charity would be rendered to them. He had, therefore, carefully examined into its affairs, as chairman of the committee, and had supported it by his personal exertions, satisfied that neither he nor any one else could render a greater good to the poor of this metropolis and the country at large.

Lord GRANTHAM said he had, as one of the building committee, looked repeatedly to the plans, and had been enabled to strike off every thing that was not purely useful, so that a well-built and well-arranged hospital for thirty persons of both sexes, with their attendants, as in-patients, and the necessary accommodation for two hundred out-patients, would be obtained for the sum of £4000. This was, however, entirely owing to the kindness and generosity of Sir Edward Banks, who had undertaken to build the hospital, and had not only done it on these terms, but had also most liberally contributed £200; and who, although he knew that the whole of the money had not been subscribed, had not hesitated to begin building, satisfied that the public would not allow such an undertaking to remain incomplete; and he had no doubt that, when its nature and objects should be fully understood, and the building seen, the necessary funds would be obtained.

Colonel Wood, M.P., the treasurer, regretted that at present the funds were not as large as he could wish: he had but £3000 subscribed, of which £600 was unpaid. He was glad to see that a part of his own subscription was in the list, because it would enable him to set the example of dunning himself, and, with the permission of the meeting, he should do so to every one else. He should want £2000 to enable him to complete the building, with its necessary furniture, so as to be fit for the reception of thirty in-patients and two hundred out-patients; and it was estimated that the sum of £800 would be sufficient, with strict economy, to meet the annual expense. The amount of expenditure in the past year had been £357. 10*s.* with only four in-patients, and he should require £500 a year more, to enable him to carry into effect his own wishes and that of the committee. He had sent up to London, from his place in the country, a poor fellow who had lost both his eyes, in a few days, from a malignant attack of inflammation: they had burst, and no hope was entertained of their recovery; by an operation, however, he had been enabled to see with one eye, so as to assist himself, and the parish was, in a great measure, relieved from a burden which might have lasted for fifty years. He had no doubt that many poor persons and parishes were similarly situated, and that the hospital, if duly supported, would afford them inestimable benefit and relief. Among other subscriptions received this day, he must particularize a third donation of £100 from Sir J. Swinburne, making in all the sum of £325 he had given to the building fund: this from a gentleman who had attended so closely to the affairs of the charity, must be its strongest recommendation to the public.

Sir James M'Grigor, the director general of the medical department of the army, was most anxious to bear testimony to the benefits derived by the poor from this charity: it was also a great school of instruction for medical students, in which they obtained knowledge they were afterwards capable of diffusing over every quarter of the globe. The officers of the public service had always been admitted gratuitously, not only to see the practice of the hospital, but to attend all the lectures on the practice of surgery delivered by the surgeon; and the institution, on these accounts, should always have his best support and recommendation.

Sir William Burnett, the medical commissioner and head of the medical department of the navy, most cordially agreed in every thing which had been said by Sir James M'Grigor. The liberal manner in which this hospital has been opened to the medical officers of the navy, was beyond all praise: many of them had greatly benefited by it, and he sincerely hoped the hospital would be soon placed on that footing, by the liberality and charity of the public, which would enable it to fulfil all the objects it had in view, and which it was so desirable should be accomplished.

Thanks were voted, on the motion of Lord Grantham, seconded by Mr. Byng, to the Duke of Richmond, who assured the governors generally of the interest he felt in the prosperity of the charity; and his grace then proceeded to lay the stone under the centre window of the ground floor of the south-west front, bearing the following inscription: "The first stone of this hospital was laid by Charles Duke of Richmond, Knight of the Garter, Postmaster General, and one of the Vice-presidents, on the 29th June, 1831."

It must be no trifling source of gratification to Mr. Guthrie to witness the interest which is taken in the completion of an institution of which he is the chief medical officer. We can add our own testimony to the observations of Sir J. Swinburne, as to the crowds of patients who have sought Mr. Guthrie's advice, and benefited by his skill, at the establishment in Warwick street; and no doubt can be entertained of the high character which the Royal Westminster Ophthalmic Hospital will obtain, under his surgical superintendence.

### NEW FEVER HOSPITAL.

AMIDST the numerous charitable institutions with which the metropolis abounds, there has hitherto been but one place of refuge for the poor and destitute, when labouring under contagious fevers, to which they could have easy and immediate admission. The Fever Hospital at Battlebridge is much too small in size for the demands that are constantly made upon it, and we have often known patients, labouring under contagious fevers, obliged to remain in their wretched and miserable habitations, at their own peril and that of all around them, because they could not obtain admission into any charitable institution: we are much gratified to find, therefore, that the Archbishop of Canterbury, the Bishops of London and Llandaff, together with Drs. BIRKBECK, BABINGTON, SOUTHWOOD SMITH, &c., have come forward, for the purpose of instituting an additional asylum for those who may be afflicted with contagious fevers. We cannot doubt that so philanthropic a project will meet with the support it so well deserves.

Subscriptions will be received at the principal banking houses.

---

### CHOLERA.

*Despatches from* Dr. RUSSEL *and* Dr. BARRY.

Dr. RUSSEL and Dr. BARRY, who had proceeded to St. Petersburgh for the purpose of obtaining the necessary authority to secure the facilities at Riga requisite for the objects of their mission, finding that cholera had made its appearance in the Russian capital, have judged it advisable to remain there, in order to watch the disease in a situation where they have an opportunity of witnessing its commencement as well as its progressive stages. The despatch was written so soon after their arrival, however, and the breaking out of the malady had been so recent, that time did not serve for more than identifying the cholera which prevailed on the shores of the Baltic with that which has been of late years the scourge of Hindoostan. This identity was at once recognized by Dr. Russel, to whom the cholera of India was familiar. Little, if any doubt, can be said to have existed on this point: still it is satisfactory to have the general opinion confirmed by one who has been an eyewitness of the disease in its two very different and distant localities. The only circumstance worthy of notice, in addition to those we have previously mentioned, is one pointed out by Dr. Russel; namely, the peculiar and characteristic sensation communicated by the tongue of the patient on applying the finger to it. In appearance it is at first but little altered, yet even then it feels cold, and exactly like touching a portion of raw flesh.

No opinion is expressed in the despatch as to the contagious or non-contagious nature of the disease. The mere fact of its manifestation in St. Petersburg, despite the triple cordon of troops, is a *prima facie* evidence against contagion: but, by a private letter which is in town, we learn that the first case occurred in a person who had come down the river in a bark; the second in an individual who had been on board after its arrival; and the third in a soldier who had mounted guard on the boat, to prevent any intercourse with those on shore. If this be the whole truth, and nothing but the truth, it is almost conclusive in favor of the contagionists; but it is to be kept in mind that the Russian authorities had previously decided that the disease was infectious, and the testimony of those who have already committed themselves by the expression of a positive opinion is to be received with some degree of caution.

The disease was rapidly spreading, and we doubt not but that the next arrivals will bring further particulars from the medical commissioners.—*Med. Gazette.*

CHOLÉRA AT WARSAW.

*Extract of a Letter from* MR. SEARLE, *dated Warsaw, July 4th.*

I AM placed in charge of an hospital for the reception of the poor, a mile and a half from the city. The first difficulty I have to cope with is, that few patients are admitted till their extremities are livid, the pulse lost at the wrist, and the evacuations have spontaneously ceased: this comes of the Medical Board of this place having circulated advice that every person should be bled on becoming the subject of the disease, and drink plentifully of hot water. This treatment is consequently considered specific, and it is not, therefore, till every individual has proved the error, that they think of coming to the hospital; to say nothing of the expense and trouble they are subjected to in coming so far. The next thing is, that, when admitted, there is nobody but a few Russian prisoners to see the remedies administered, or to attend upon the sick, except a couple of dressers, who invariably leave the hospital the moment my back is turned; that, in short, nothing is done for them but what is done by myself in person. I have represented the thing to the heads of the department over and over again; promise has been made me upon promise, and in this way have I been fooled up to the time present. Yesterday I made a strong and pressing communication to the government on the subject: what the result will be, I know not. The true state of the case is this, that the Council of Medicine, consisting of a dozen private practitioners of the place, are a junto (for the most part) of ignorant men, and extremely jealous of us foreigners, both English and French, and, I believe, do their utmost to thwart us in every way. I say us, as, although they make great professions of respect and good feeling towards me, yet they do nothing that I require, but only promise. This may be well denominated the land of promises, for these they make in profusion; and it is the same yesterday, today, and for ever. Never, in any situation that I have filled through life, did I feel so truly humbled and disgusted. I feel resources within myself, and burn to exercise them towards the poor and suffering creatures: but no, I am constrained to stand little better than an idle spectator of misery and death, which it is in my power to alleviate, but am not permitted to do so.

The disease here, which is precisely the same in character with that in India, is unquestionably, as I have represented, of the febrile class, and dependent upon the same causes as the typhoidal orders of fever usually are, marshy and pestiferous exhalations, arising in low, damp, filthy, unventilated situations. To the developement of the disease, or to render the individual susceptible of its attack, it would, however, appear that a certain condition of system or predisposition must exist, or it would be of more general influence where filth of every kind so much abounds as it does here. This condition I believe to consist simply or principally in debility: hence the indifferently fed, the badly clothed, and comfortless poor, or those exposed to the inclemencies of the weather and vicissitudes of temperature, and particularly under exhaustion from want of food and bodily fatigue, are the most frequent subjects of its attack. The treatment I find most successful corresponds with the advice given in my work: evacuating the stomach, in the first instance, with a solution of *salt*, in the proportion of a large tablespoonful to a tumbler of hot water: this operates almost instantly as an emetic; or where, from atony of stomach, it does not so, a second is administered. The patient, then, for cleanliness' sake, is sponged all over with one pint of nitric acid, diluted with two of hot water, and, hot flannels being in readiness, he is well rubbed with them for half an hour; when, being placed in a warm bed, in an airy room, five grains of calomel are administered every half hour, with a spoonful of brandy added to two of hot water. As excitement becomes developed, the spirit is diminished in quantity, and the intervals between each dose of calomel prolonged, but continued till bilious stools and urine are obtained. Should the breathing be much oppressed, or the fulness of the præcordia, or oppression of the brain considerable, bleeding is required, but not in too large a quantity. The fever which generally follows, being of the typhoidal character, requires a great deal of nice management, and they almost invariably die here from neglect: it is always attended with some organic inflammation, which is not to be cured by evacuants. This fever is, however, to be prevented, I am of opinion, by carrying on the calomel to the production of a tender state of the gums, or gentle ptyalism: but my views are, instead of persevering with the calomel to this extent, to follow up the treatment, on the appearance of bilious evacuations and urine, (or the restoration of secretion,) by the quinine, or bark and wine.

I have just completed an apparatus for the inhalation of chlorine, which I intend giving a trial to. The nitrous oxide, or oxygen gas, I should have preferred; but there are difficulties attending these which, I fear, are insurmountable.

I think it by no means improbable you will have the disease, or something of the kind, in England, as I cannot help connecting its appearance in Europe with some peculiar epidemic influence of atmosphere, seeing that, when I passed through Berlin, there was an epidemic fever prevailing there, with which 40,000 persons were attacked out of a population of 200,000, and hearing since then that an influenza, or something of the sort, is prevailing in Paris.

The connexion between cholera and fever has been remarkably exemplified here: at least I have been told that intermittent fever was prevailing to some extent before the cholera; on this appearing, the former vanished, and reappeared on the cessation of cholera. The weather here is the most unpleasant I have ever experienced: for a few days it was so hot that I never felt it so oppressive, that I remember of, all the time I was in India; this has been followed by constant rain; and with the few comforts the country affords us, it is truly wretched.—*Ibid.*

### LITERARY INTELLIGENCE.

Mr. Waller has in the press a second edition of his "Elements of Practical Midwifery," which contains an additional quantity of matter, and will be published by S. Highley, Fleet street, in a few days.

Mr. Charles Severn will shortly publish "First Lines of the Practice of Midwifery, with Remarks on Fœticide, Infanticide, and the Forensic Evidence requisite in proof of the commission of those crimes."

### METEOROLOGICAL JOURNAL,

By Messrs. HARRIS and Co. Mathematical Instrument Makers, 50, High Holborn.

| | Moon. | Rain gauge. | Thermom. | | | Barometer. | | De Luc's Hygrometer. | | Winds. | | Atmospheric Variation. | | |
|---|---|---|---|---|---|---|---|---|---|---|---|---|---|---|
| June | | | 9 a.m. | max. | min. | 9 a.m. | 10 p.m. | 9 a.m. | 10 p.m. | 9 a.m. | 10 p.m. | 9 a.m. | 2 p.m. | 10 p.m. |
| 20 | | | 68 | 72 | 58 | 29.98 | 30.00 | 47 | 46 | WSW | W | Fine | Fine | Fine |
| 21 | | | 67 | 70 | 60 | 30.00 | .00 | 46 | 46 | WSW | WSW | — | — | — |
| 22 | | | 69 | 73 | 61 | .01 | .01 | 46 | 47 | NW | N | — | — | — |
| 23 | | | 69 | 69 | 59 | .01 | 29.92 | 47 | 47 | NE | W | — | — | Cloudy |
| 24 | | | 61 | 64 | 59 | 29.75 | .66 | 47 | 46 | WSW | NW | Sho'ry | — | Fine |
| 25 | ○ | | 64 | 64 | 53 | .58 | .48 | 46 | 47 | NW | W | Cloudy | Cloudy | Sho'ry |
| 26 | | | 58 | 61 | 54 | .58 | .58 | 49 | 55 | NW | N | Sho'ry | Sho'ry | Cloudy |
| 27 | | .21 | 60 | 62 | 55 | .71 | .61 | 52 | 54 | SW | SSW | Fine | Fine | Rain |
| 28 | | | 57 | 60 | 56 | .72 | .82 | 54 | 54 | NNE | N | Cloudy | Cloudy | Cloudy |
| 29 | | .28 | 58 | 62 | 56 | .83 | .83 | 54 | 55 | N | NW | — | — | — |
| 30 | | | 58 | 65 | 57 | .85 | .88 | 55 | 51 | NNW | NNE | — | — | — |
| July. 1 | | | 65 | 65 | 54 | .91 | .86 | 49 | 48 | N | NNE | Fine | Fine | Fine |
| 2 | ☾ | | 58 | 63 | 57 | .82 | .80 | 48 | 48 | NW | WSW | — | — | — |
| 3 | | | 66 | 71 | 60 | .86 | .98 | 47 | 47 | SW | W | — | Cloudy | — |
| 4 | | | 66 | 70 | 62 | 30.01 | 30.02 | 47 | 47 | WNW | SW | — | Fine | — |
| 5 | | | 68 | 71 | 62 | .02 | .04 | 47 | 48 | W | WSW | Cloudy | — | — |
| 6 | | | 70 | 70 | 58 | .14 | .16 | 50 | 49 | NE | ESE | Fine | — | — |
| 7 | | | 68 | 69 | 60 | .13 | .07 | 49 | 50 | ESE | ESE | — | — | — |
| 8 | | | 65 | 70 | 62 | .03 | 29.98 | 50 | 48 | E | NE | Cloudy | — | — |
| 9 | ● | | 71 | 76 | 66 | 29.93 | .91 | 45 | 45 | N | E | Fine | — | — |
| 10 | | .27 | 72 | 79 | 59 | .82 | .74 | 48 | 51 | NW | W | — | Rain | Cloudy |
| 11 | | | 67 | 70 | 62 | .69 | .58 | 50 | 50 | N | NNW | — | Fine | — |
| 12 | | | 68 | 69 | 59 | .45 | .46 | 49 | 54 | W | ENE | Cloudy | Cloudy | — |
| 13 | | .33 | 60 | 64 | 57 | .42 | .50 | 61 | 59 | SE | S | Rain | Fine | — |
| 14 | | .58 | 63 | 63 | 57 | .54 | .54 | 55 | 57 | S | S | Rain | Cloudy | — |
| 15 | | .70 | 61 | 65 | 58 | .54 | .59 | 57 | 56 | W | SSW | — | Rain | — |
| 16 | ☽ | | 64 | 69 | 59 | .54 | .61 | 56 | 54 | SSE | WNW | Sho'ry | Cloudy | Fine |
| 17 | | .16 | 68 | 69 | 59 | .78 | .83 | 54 | 54 | NW | SW | Fine | Fine | — |
| 18 | | | 66 | 67 | 58 | .81 | .72 | 53 | 52 | W | WSW | — | — | Cloudy |
| 19 | | | 62 | 64 | 60 | .72 | .69 | 52 | 53 | W | SW | — | — | — |

The quantity of Rain fallen, in the month of June, was 1 inch and 89.100ths.

